# Herpes simplex virus lymphadenitis is associated with tumor reduction in a patient with chronic lymphocytic leukemia

**DOI:** 10.1172/JCI161109

**Published:** 2022-09-15

**Authors:** Andres Chang, Anton M. Sholukh, Andreas Wieland, David L. Jaye, Mary Carrington, Meei-Li Huang, Hong Xie, Keith R. Jerome, Pavitra Roychoudhury, Alexander L. Greninger, Jean L. Koff, Jonathon B. Cohen, David M. Koelle, Lawrence Corey, Christopher R. Flowers, Rafi Ahmed

**Affiliations:** 1 Department of Hematology and Medical Oncology, Winship Cancer Institute and; 2 Emory Vaccine Center, Department of Microbiology and Immunology, Emory University School of Medicine Atlanta, Georgia, USA.; 3 Vaccine and Infectious Disease Division, Fred Hutchinson Cancer Research Center, Seattle, Washington, USA.; 4 Departments of Laboratory Medicine and Pathology, University of Washington, Seattle, Washington, USA.; 5 Department of Pathology and Laboratory Medicine, Emory University School of Medicine, Atlanta, Georgia, USA.; 6 Basic Science Program, Frederick National Laboratory for Cancer Research, and Laboratory of Integrative Cancer Immunology, Center for Cancer Research, National Cancer Institute, Bethesda, Maryland, USA.; 7 Ragon Institute of MGH, MIT and Harvard, Boston, Massachusetts, USA.; 8 Department Medicine and; 9 Department of Global Health, University of Washington, Seattle, Washington, USA.; 10 Department of Translational Immunology, Benaroya Research Institute, Seattle, Washington, USA.

**Keywords:** Hematology, Immunology, Adaptive immunity, Cancer immunotherapy, Leukemias

## Abstract

**Background:**

Herpes simplex virus lymphadenitis (HSVL) is an unusual presentation of HSV reactivation in patients with chronic lymphocytic leukemia (CLL) and is characterized by systemic symptoms and no herpetic lesions. The immune responses during HSVL have not, to our knowledge, been studied.

**Methods:**

Peripheral blood and lymph node (LN) samples were obtained from a patient with HSVL. HSV-2 viral load, antibody levels, B and T cell responses, cytokine levels, and tumor burden were measured.

**Results:**

The patient showed HSV-2 viremia for at least 6 weeks. During this period, she had a robust HSV-specific antibody response with neutralizing and antibody-dependent cellular phagocytotic activity. Activated (HLA-DR^+^, CD38^+^) CD4^+^ and CD8^+^ T cells increased 18-fold, and HSV-specific CD8^+^ T cells in the blood were detected at higher numbers. HSV-specific B and T cell responses were also detected in the LN. Markedly elevated levels of proinflammatory cytokines in the blood were also observed. Surprisingly, a sustained decrease in CLL tumor burden without CLL-directed therapy was observed with this and also a prior episode of HSVL.

**Conclusion:**

HSVL should be considered part of the differential diagnosis in patients with CLL who present with signs and symptoms of aggressive lymphoma transformation. An interesting finding was the sustained tumor control after 2 episodes of HSVL in this patient. A possible explanation for the reduction in tumor burden may be that the HSV-specific response served as an adjuvant for the activation of tumor-specific or bystander T cells. Studies in additional patients with CLL are needed to confirm and extend these findings.

**Funding:**

NIH grants 4T32CA160040, UL1TR002378, and 5U19AI057266 and NIH contracts 75N93019C00063 and HHSN261200800001E. Neil W. and William S. Elkin Fellowship (Winship Cancer Institute).

## Introduction

Herpes simplex virus 2 (HSV-2) is a sexually transmitted infection that establishes a chronic latent infection in the associated dorsal root ganglion, with episodes of reactivation and viral shedding that are asymptomatic or result in typical, localized herpetic lesions (1, 2). However, in patients with chronic lymphocytic leukemia (CLL), HSV reactivation may not lead to the development of herpetic lesions but instead can result in HSV lymphadenitis (HSVL). Patients with HSVL usually present with fever, night sweats, and worsening lymphadenopathy (3). Because of these signs and symptoms and the absent herpetic lesions, HSVL is often mistaken as aggressive lymphoma transformation. The pathophysiology of this unusual and challenging diagnosis is unknown but is not associated with CLL subtype or therapy (4). It is hypothesized that immunological defects caused by CLL contribute to this manifestation, but, to our knowledge, the immune responses in HSVL have not been examined. Here, we present a detailed analysis of the HSV-specific adaptive immune responses in a patient with CLL who had recurrent HSVL, and we report the concomitant presence of viremia caused by a typical HSV-2 strain and describe a sustained decrease in tumor burden after each occurrence.

### Patient case description.

**Figure 1:**
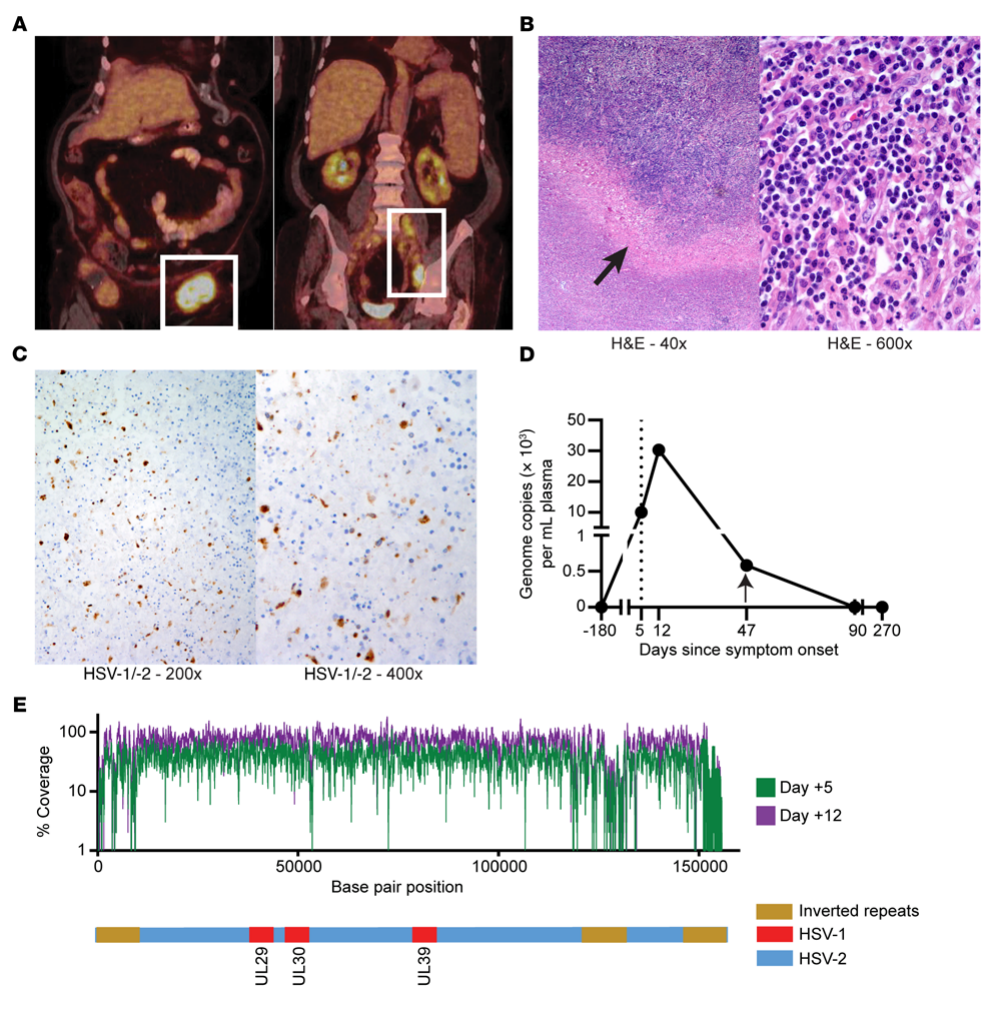
Clinical presentation of HSVL in a patient with CLL. (**A**) A PET/CT scan obtained at presentation showed left-sided FDG-avid adenopathy. (**B**) Low- and high-power micrographs of H&E staining showing areas of necrosis (arrow) in the biopsy specimen of the FDG-avid left obturator node. Original magnification, ×40 (left) and ×600 (right). (**C**) Cells stained for HSV-1/-2 major capsid protein were found in the biopsy sample (brown). Original magnification, ×200 (left) and ×400 (right). (**D**) Plasma HSV-2 genome copies over time showing evidence of HSV-2 viremia. Vertical dotted line indicates the time of presentation; arrow indicates the time of antiviral therapy initiation. (**E**) Top: Sequence coverage depth for plasma-derived HSV-2 DNA from day +5 (green) and day +12 (purple). Coordinates are from the HSV-2 reference strain HG52 (GenBank accession JN561323.2). Poor coverage of UL and US inverted repeats is commonly noted with short-read technologies. Bottom: Schematic of HSV-2 genomes showing inverted repeats and HSV-1 insertions in 3 indicated HSV ORFs that are prevalent in North American strains (5) and that were detected in both patient specimens.

A 79-year-old woman with treatment-naive CLL (del13q and trisomy 12) presented approximately 7 years after diagnosis with a 5-day history of fatigue, malaise, and tender, rapidly enlarging left inguinal adenopathy but no mucocutaneous lesions. Laboratory testing revealed new-onset anemia and thrombocytopenia but normal lactate dehydrogenase (LDH). A bone marrow biopsy showed CLL involvement with no evidence of aggressive lymphoma. Fluorodeoxyglucose PET/CT (FDG-PET/CT) showed retroperitoneal and left iliac adenopathy and a left groin mass measuring 7.4 × 4.3 cm with a maximum standardized uptake value (SUV) of 14.3 (Figure 1A). Excisional biopsy of the mass 17 days after symptom onset revealed a lymph node (LN) involved by CLL with prominent necrotic areas but no aggressive lymphoma (Figure 1B). Prior reports indicated that HSV reactivation in patients with CLL can, in rare instances, cause these symptoms (4). Thus, to confirm this diagnosis, we performed immunohistochemical staining for HSV-1/-2, which showed HSV-infected cells (Figure 1C). PCR of LN material was also positive for HSV-2. The patient was treated with corticosteroids and valacyclovir followed by acyclovir prophylaxis, with complete symptom resolution and no recurrence for over 3 years.

## Results

### HSV-2 viremia and viral sequence.

The availability of baseline and longitudinal samples collected within 270 days of presentation (Supplemental Figure 1; supplemental material available online with this article; https://doi.org/10.1172/JCI161109DS1) allowed us to study the virology of and immunological responses in HSVL. Plasma real-time quantitative PCR revealed HSV-2 viremia, with detectable viral DNA for at least 6 weeks (Figure 1D). High-resolution sequencing recovered more than 99% of known protein-coding regions of the HSV-2 genome from the blood on days +5 and +12, which showed no significant differences between samples, displayed high similarities to common US strains, and showed no genotypic evidence of acyclovir resistance (Figure 1E) (5, 6). These findings indicate that reactivation of a conventional HSV-2 strain, and not a distinct HSV-2 variant, produced a systemic infection in this patient.

### HSV-specific antibody responses in HSVL.

**Figure 2:**
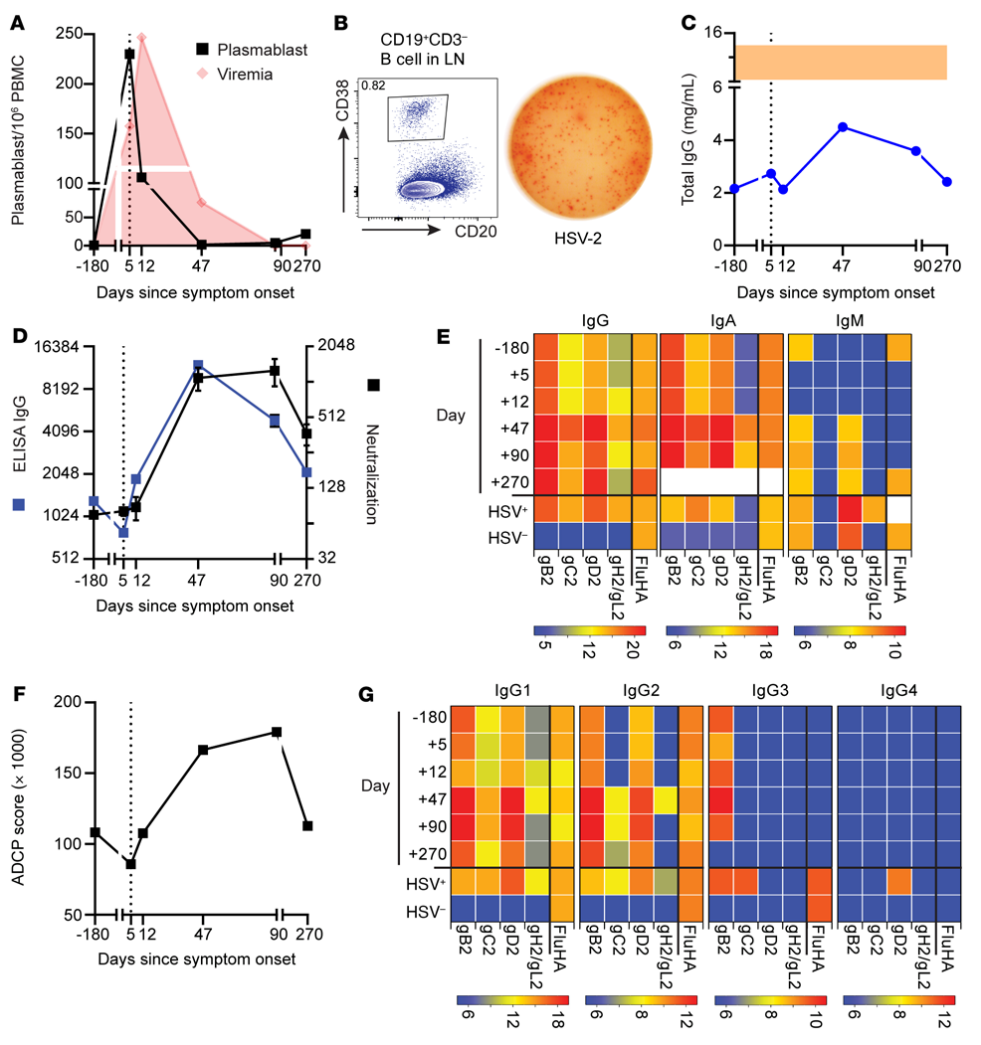
Vigorous HSV-specific B cell responses were observed in the blood and LN. (**A**) Quantification of plasmablasts per million live PBMCs in the blood over time. Red indicates the viral titer depicted in Figure 1C. (**B**) Flow cytometric analysis shows plasmablasts in the LN (left). ELISPOT assay (right) shows that LN plasmablasts secreted HSV-2–specific IgG antibodies. (**C**) Persistent hypogammaglobulinemia was observed in this patient. Shaded area indicates the expected normal range. (**D**) Relative IgG-binding titers against HSV-2 lysates by ELISA (blue) and neutralizing antibody titers leading to a 50% reduction in virus infectivity (black). Error bars indicate the SEM. Positive control neutralizing antibody titer = 1:256. (**E**) IgG, IgA, and IgM antibody binding to different HSV-2 surface proteins at each time point versus pooled plasma from HSV-2^+^ and HSV-1/-2^–^ controls. White denotes the sample not analyzed. (**F**) HSV-2 ADCP score (in thousands) of gD2-covered microspheres incubated with plasma from each time point. ADCP score for pooled healthy HSV-2^+^ control = 111,320. Error bars indicate the SD. (**G**) IgG subclass analysis of antibodies that bound to HSV-2 surface proteins at each time point versus pooled plasma from HSV-2^+^ and HSV-1/-2^–^ controls. For **E** and **G**, antibody binding to influenza HA (FluHA) was included as a control. Heatmap scale reflects the endpoint titer in log_2_. All measurements were performed in duplicate. For all applicable graphs, the vertical dotted line indicates the time of presentation. Day, days since symptom onset.

Patients with CLL are known to have a dysregulated immune system (7), but the immunological factors that allowed for this patient’s clinical presentation were unknown. Thus, we set out to further evaluate the HSV-specific adaptive immune responses in our patient. Flow cytometric analysis (FACS) of PBMCs detected higher numbers of antibody-secreting cells (plasmablasts), defined as CD19^+^CD3^–^CD20^–/lo^CD38^hi^IgD^–^ lymphocytes, at presentation compared with other time points (Figure 2A) (8). Plasmablasts lacked CD5 expression, indicating that these were not CLL cells. Assessment of the LN sample obtained on day +17 also showed high numbers of plasmablasts by FACS (Figure 2B, left). Using an ELISPOT assay, we confirmed that plasmablasts in the LN secreted IgG antibodies that recognized HSV-2 (Figure 2B, right) (8).

Despite the patient’s persistent hypogammaglobulinemia (Figure 2C), as is often seen in CLL, HSV-2–specific IgG titers were detectable at baseline, started to increase by day 12, and peaked after 6 weeks, resulting in a 14-fold increase (*P =* 0.0015, Figure 2D). These antibodies reacted against the 4 major HSV-2 glycoproteins (gB2, gC2, gD2, and the gH2/gL2 heterodimer) and remained elevated for at least 270 days (Figure 2E). Increases in HSV-2–specific IgA titers were similar to those of IgG, whereas IgM titers only increased 1- to 2-fold (Figure 2E).

To determine the functional capacity of these antibodies, we measured their neutralizing potential and their capacity to elicit antibody-dependent cellular phagocytosis (ADCP). Neutralizing antibody titers increased 13.5-fold by week 6 (Figure 2D). ADCP capacity against gD2-covered microspheres also increased, peaking at 90 days (Figure 2F). We also measured IgG subclasses of the HSV-2–specific antibodies and found a predominant IgG1 response (Figure 2G), consistent with ADCP and neutralizing activity. We also observed significant IgG2 and IgG3 responses. IgG1 and IgG3 antibodies have potent Fc effector functions and usually increase in response to viral infections (9). We also detected changes in IgA and IgM levels. Overall, our findings indicate that this patient with CLL mounted a robust and functional humoral immune response after HSV reactivation.

### Activation of CD4^+^ and CD8^+^ T cells in response to HSVL.

**Figure 3:**
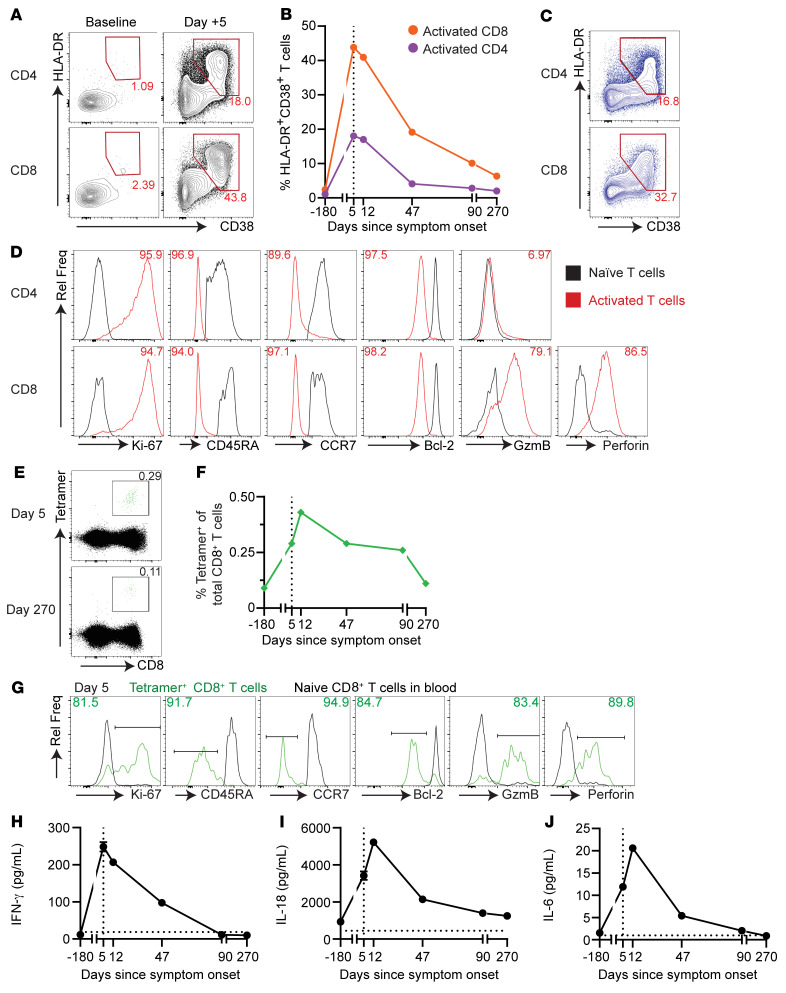
Robust T cell and plasma cytokine responses were observed. (**A**) FACS plots of singlet, live, CD3^+^, CD4^+^, and CD8^+^ lymphocytes at baseline and on day +5, identifying activated HLA-DR^+^CD38^+^ T cells. (**B**) Percentage of activated CD4^+^ (purple) and CD8^+^ (orange) T cells over time as analyzed in **A**. (**C**) FACS analysis showing a high percentage of activated CD4^+^ and CD8^+^ T cells in the LN. Numbers in **A** and **C** denote the percentage of total CD4^+^ or CD8^+^ T cells. (**D**) CD38^+^HLA-DR^+^ T cells in the blood (red) had an effector phenotype. Numbers denote a percentage of activated CD4^+^ or CD8^+^ T cells expressing or downregulating the marker of interest. Naive (black) CD4^+^ or CD8^+^ T cells are shown as a control. All gates were set in reference to naive CD4^+^ or CD8^+^ T cells. (**E**) UL-25 tetramer staining in the blood on day +5 and day +270. Numbers denote the percentage of total CD8^+^ T cells. (**F**) Percentage of HSV-2 UL-25–specific tetramer^+^CD8^+^ T cells in the blood over time. (**G**) Extended phenotype analysis of UL-25 tetramer^+^CD8^+^ T cells in the blood on day 5. Green indicates UL-25 tetramer^+^CD8^+^ T cells; black indicates naive CD8^+^ T cells. Numbers indicate the percentage of tetramer^+^ (green) CD8^+^ T cells. Mean plasma levels of proinflammatory cytokines IFN-γ (**H**), IL-18 (**I**), and IL-6 (**J**) were elevated in the acute setting. All measurements were performed in quadruplicate. Error bars indicate the SD. Horizontal dotted line indicates the levels in pooled plasma from aged-matched healthy individuals. For all applicable graphs, the vertical dotted line indicates the time of presentation. Day, days since symptom onset. Rel Freq, relative frequency.

T cell responses were also characterized, given their important role in controlling HSV (10). We observed an 18-fold increase in the frequency of activated CD4^+^ and CD8^+^ T cells at presentation compared with baseline frequencies (18% and 43.8% of total CD4^+^ and CD8^+^ T cells, respectively), as defined by coexpression of HLA-DR and CD38 (Figure 3, A and B). Activated CD4^+^ and CD8^+^ T cells were markedly less numerous in the blood by 6 weeks (Figure 3B). High numbers of activated CD4^+^ and CD8^+^ T cells were also observed in the LN (Figure 3C). To further characterize these cells, we analyzed several markers associated with effector T cell differentiation (11). Consistent with an effector phenotype, activated T cells showed high levels of Ki-67 (indicating active proliferation). CD8^+^ T cells also expressed high levels of perforin and granzyme B (GzmB), indicating cytotoxic ability (Figure 3D). These cells also showed lower expression of the antiapoptotic protein Bcl-2 and lacked CD45RA and CCR7, consistent with an effector CD8^+^ T cell phenotype.

To further characterize the HSV-2–specific CD8^+^ T cell response, we generated an MHC class I (MHC-I) tetramer using an epitope known to be presented by an MHC-I allele similar to the patient’s allele (Figure 3E) (12). CD8^+^tetramer^+^ cells were found in the blood at all time points and expanded 4-fold by day +12 (Figure 3F). CD8^+^tetramer^+^ cells on day +5 also showed an effector phenotype (Figure 3G). We also detected CD8^+^tetramer^+^ cells in the LN (data not shown). These data demonstrate that the patient had HSV-specific CD8^+^ T cells at baseline and mounted a robust CD4^+^ and CD8^+^ T cell response in the blood and LN upon HSV reactivation.

The levels of proinflammatory cytokines were also measured. We observed a 21-fold increase in IFN-γ levels at presentation compared with baseline levels (Figure 3H). IL-18 levels also increased (Figure 3I). IL-18 is a cytokine that can enhance antigen-independent IFN-γ production by effector and memory CD8^+^ T cells, improving their activity (13). Plasma levels of other proinflammatory cytokines such as IL-6 (Figure 3J), TNF-α, IP-10, IL-27, IL-1RA, IL-22, and MIP-1α (Supplemental Figure 2) were also markedly increased at presentation. Proinflammatory cytokine levels returned to baseline around the time the patient’s symptoms resolved. These results indicated a profound proinflammatory state in this patient during the viremic stage.

### Association between HSVL and decreased CLL tumor burden.

**Figure 4:**
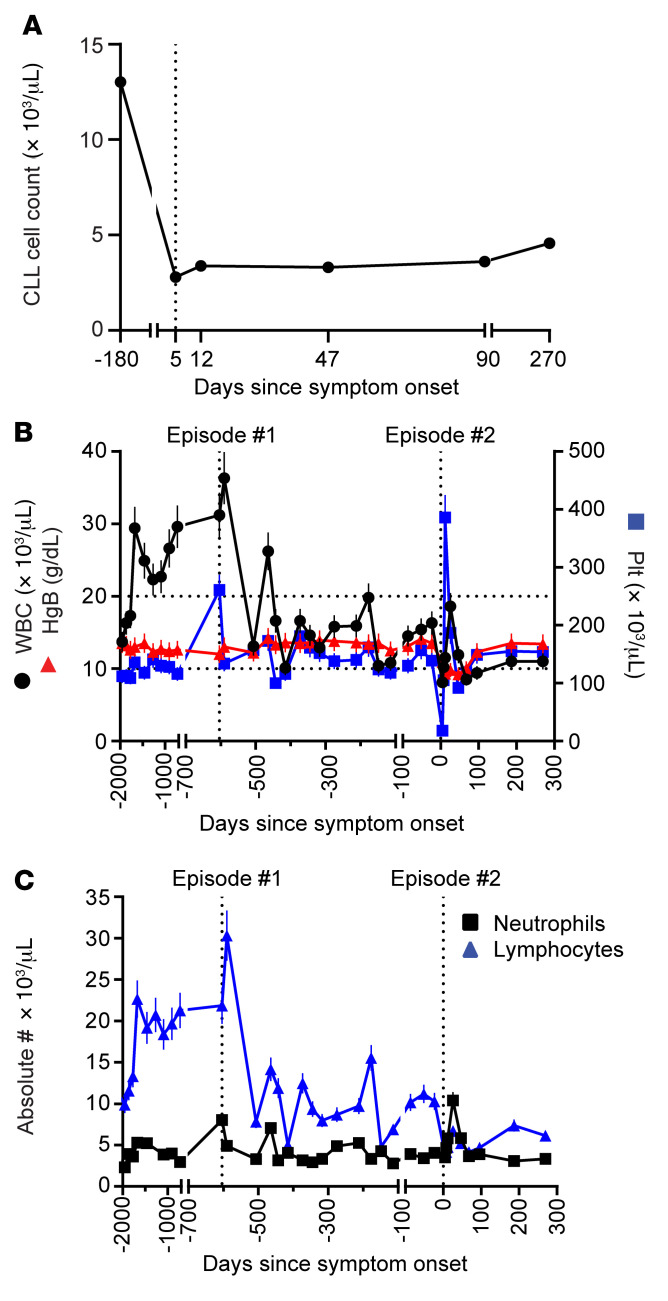
Decreased circulating CLL tumor burden after HSVL. (**A**) Absolute CLL cell count (in thousands) per μL blood as assessed by FACS. (**B**) WBC (black), hemoglobin (HgB, red), and platelet (Plt, blue) counts over time showed a sustained decrease in the absolute leukocyte count after each HSVL episode. Vertical dotted lines represent values at the first and second presentations of an HSVL episode. Horizontal dotted lines indicate the WBC count ranges between the first and second HSVL episodes. (**C**) Absolute neutrophil (black) and lymphocyte (blue) counts over time. The decrease in WBCs was largely due to a decrease in the absolute lymphocyte count (blue) after each HSVL episode, as the absolute neutrophil (black) count remained constant. For **B** and **C**, error bars indicate the SD.

Interestingly, and surprisingly, we observed a significant reduction in circulating CLL cell numbers at presentation, which remained stable without the use of CLL-directed therapies, even after resolution of the viremia (Figure 4A). Moreover, further review of the patient’s records showed that she experienced a similar episode 2 years prior that also resulted in a significant and sustained reduction in her WBC count (Figure 4B, episode 1). This decrease in WBCs was mostly attributed to a decrease in lymphocytes, as her neutrophil counts remained largely stable (Figure 4C). At that time, she presented with similar symptoms including rapidly enlarging right inguinal adenopathy. FDG-PET/CT revealed FDG-avid retroperitoneal, right iliac, and inguinal lymphadenopathy as well as a 5.1 × 2.1 cm right obturator LN with a SUV of 11.3 (Supplemental Figure 3A). Excisional biopsy of this LN at that time showed CLL, prominent necrosis, and no aggressive lymphoma (Supplemental Figure 3B), so the patient was administered corticosteroids, which led to symptom resolution. We retrieved the patient’s archived LN, and staining showed HSV-1/-2^+^ cells (Supplemental Figure 3C), establishing her initial diagnosis of HSVL and documenting the subsequent disease recurrence. Our findings therefore showed sustained tumor control after each episode of HSVL.

## Discussion

This study provides several insights into the clinical presentation and breadth of the adaptive immune responses in HSVL, with the key finding that each episode of HSVL in this patient was associated with reductions in CLL cell counts lasting long after resolution of the acute process. Clinically, the finding of viremia in the acute setting is intriguing, since HSV viremia is rare, and the patient had no herpetic lesions. The presence of HSV viremia in this patient suggests that HSVL could be a manifestation of a systemic HSV infection. Given the similarity in clinical presentations between HSVL and aggressive lymphoma transformation, testing for HSV viremia during workup could facilitate its prompt diagnosis and treatment and help elucidate its true incidence, as fewer than 50 cases have been reported (14). FACS showing prominent T cell activation could also help support the diagnosis of HSVL. Nonetheless, further studies are needed to determine whether these tests, combined with localized photopenia on FDG-PET/CT (15, 16) in the appropriate clinical context, could be diagnostic for HSVL and thus prevent unnecessary invasive procedures.

The pathophysiology of HSVL remains unclear, but the identification of a conventional HSV-2 strain in this patient suggests that host factors, rather than variations in HSV viral sequences, were primarily responsible for this unusual clinical presentation of HSV reactivation. A robust humoral and T cell response in the blood and LN was observed in this episode of HSVL. Notably, even though patients with CLL are known to have IgG deficiencies (17) and our patient had low total IgG levels throughout the analysis period, we found that baseline IgG levels against some HSV-2 surface glycoproteins were equivalent to healthy HSV-2–seropositive control levels and that titers against most of these proteins were substantially increased shortly after the acute phase. These observations contrast with the antibody responses observed after local reactivation in otherwise healthy individuals, in whom no measurable changes in circulating antibody titers were detected (18).

The interesting observation that the patient’s circulating tumor burden decreased, establishing a new, lower baseline after each HSVL episode without any CLL-directed therapies, suggests that the immune response triggered by this systemic infection helped control the CLL. Indeed, the phenomenon of tumor control after a severe infection served as early evidence of the immune system’s ability to control cancer (19–21). One potential explanation is that the systemic HSV-2 reactivation experienced by this patient was serving as an “adjuvant” for tumor-specific T cells. Consistent with this, recent studies showed that cell death induced by virus-like particles can generate potent proimmunogenic conditions and result in prolonged tumor control in a T cell–dependent manner (22). Given that HSV-2 can infect certain B cells in vitro (23), it is conceivable that CLL cell death after infection could also generate such proimmunogenic conditions, promoting sustained tumor reduction. In addition, several reports have shown that the immune responses triggered by a systemic viral infection can result in the activation of “bystander” CD8^+^ T cells present in the tumor microenvironment, resulting in delayed tumor progression (24–27). Thus, some bystander effect of HSV-2–specific T cells could have also contributed to the decrease in tumor burden. Activation of tumor-specific or bystander CD8^+^ T cells could be further enhanced by the high levels of cytokines like IL-18, augmenting antigen-independent IFN-γ production (13) and creating a more proinflammatory environment. Aside from its immunostimulatory effects, IFN-γ can also reprogram nurse-like cells to produce a less supportive tumor microenvironment (28). Elevated IL-6 levels, on the other hand, can slow leukemia progression by antagonizing TLR signaling (29). Other mechanisms including reinvigoration of CLL-specific or other virus-specific T cells by proinflammatory cytokines or a common event that simultaneously triggered both tumor regression and HSV-2 reactivation leading to HSVL are also possibilities. Although single-patient studies such as this preclude the identification of a specific mechanism that explains our patient’s clinical presentation and disease course, our study provides some insight into the pathogenesis and immunobiology of HSVL as well as evidence that virus-specific immune responses can promote sustained control of cancer cells. Further research involving larger numbers of patients is needed to better understand the effect of infections and the ensuing immune response on cancer control.

## Methods

### Patient samples.

All peripheral blood samples were collected in sodium citrate CPT tubes (BD), and PBMCs were isolated according to the manufacturer’s protocol. The patient’s plasma and PBMCs were stored at –80°C and in liquid nitrogen, respectively, until analysis. The LN sample was collected and processed as described previously (30).

### Antibodies, cell lines, viruses, and proteins.

The antibodies used in this study are listed in Supplemental Table 1. The following cell lines were used: HaCaT keratinocytes (Addex Bio) and THP-1 (American Type Culture Collection [ATCC], TIB-202). The recombinant HSV-2 strain 333, which encodes the β-gal bene (HSV-2/Gal) under CMV promoter control, inserted between virus UL3 and UL4 genes, was provided by Patricia Spear and Richard Longnecker (Northwestern University, Evanston, Illinois, USA) (31). The stem of influenza hemagglutinin (Flu-HA) strain H1 1999 NC was expressed using the vector VRC-3925 (32) in 293F cells and was provided by M. Gray and L. Stamatatos (Fred Hutchinson Cancer Research Center, Seattle, Washington, USA). The HSV-2 proteins gB2, gC2, and gH2/gL2 were provided by G. Cohen and R. Eisenberg (University of Pennsylvania, Philadelphia, Pennsylvania, USA). The HSV-2 proteins UL19ud, UL25, and gD2 (33) were provided by Immune Design Corp.

### Immunohistochemistry.

Immunohistochemistry was performed on formalin-fixed, paraffin-embedded LN biopsy samples using anti-HSV1/-2 ICP5 major capsid protein antibodies (clone 3B6; Abcam). Bound antibody was detected using EnVision FLEX+ secondary reagents on a Dako Autostainer Link48 per the manufacturer’s instructions.

### Real-time quantitative PCR.

DNA was extracted from 100 μL serum using the QIAamp 96 DNA HT kit (QIAGEN) and eluted into 100 μL AE buffer (10 mM Tris-Cl, 0.5 mM EDTA; pH 9.0). Extracted DNA (10 μL) was used to perform HSV quantitative real-time PCR (34).

### HSV-2 sequencing.

HSV-2 was sequenced from 2 PCR-positive samples using hybridization-based target enrichment and next-generation sequencing (5). Genomes were assembled using a previously described pipeline (35) and deposited in GenBank as MT461026 (day +5, 2020-3449AC) and MT461027 (day +12, 2020-3450AC). Whole-genome sequences for the 2 samples were aligned against reference sequences from GenBank (36) using MAFFT, version 7.450 (37). UL-US regions were extracted and concatenated in Geneious Prime, version 2020.1.2 (Biomatters).

### Flow cytometry.

FACS analysis was performed using either fresh or cryopreserved PBMCs. Cells were incubated with the appropriate surface antibody mix for 15 minutes, and then 1× FACS/Lyse (BD) was added to each sample. After a 10-minute incubation, the sample was washed once, permeabilized with a FOXP3 buffer kit, and stained for intracellular markers following the manufacturer’s protocol (eBiosciences). Cells were then washed once with 1× FOXP3 wash buffer followed by 1× PBS supplemented with 2% FBS and 1 mM EDTA prior to sample analysis on a BD LSR II instrument.

### Melon gel IgG isolation and total IgG concentration measurement.

Melon gel (Thermo Fisher Scientific) was used to isolate IgG from plasma according to the manufacturer’s instructions. Briefly, plasma was buffer exchanged into Melon Gel Purification buffer using Zeba Desalting spin columns (Thermo Fisher Scientific) and loaded onto Melon Gel. The flowthrough fraction containing IgG was buffer exchanged into PBS using Zeba spin columns. The final IgG preparation was sterilized, and the IgG concentration was measured using NanoDrop 200 (Thermo Fisher Scientific).

### ELISA.

HSV-2 antigens (diluted 1:100; Meridian Lifesciences) were coated on ELISA plates overnight. Plates were then washed, incubated with serum from the patient, and virus-specific IgG was detected as previously described (9).

### ELISPOT.

ELISPOT plates were coated with HSV-2 or VZV antigens (control) overnight. To assess for the presence of HSV-2–specific IgG-secreting plasmablasts in the LN, mononuclear cells were incubated in the ELISPOT plate for 18 hours, and secreted antibodies were analyzed as reported previously (8).

### HSV-2–binding antibody assay.

HSV-2 proteins and control antigens were coupled to MagPlex beads (Luminex) using the Antibody Coupling kit (Luminex) according to the manufacturer’s recommendations. Beads were blocked with PBS (Gibco, Thermo Fisher Scientific) containing 5% Blotto (Bio-Rad) and 0.05% Tween 20 (MilliporeSigma), and then incubated with serially diluted plasma samples. Pooled sera from HSV-2^+^ and HSV-2^–^ donors were included as positive and negative controls, respectively. Beads were washed and incubated with subclass-specific secondary antibodies conjugated to phycoerythrin (all from Southern Biotech). Beads were then washed and resuspended in PBS with 1% BSA and 0.05% Tween 20, and binding data were collected on a Luminex 200 instrument operated by MagPlex software (Hitachi). Bead regions were detected, and fluorescence was measured for at least 70 beads per region to calculate the MFI for each region. The MFI measured with unconjugated beads was considered the background and subtracted from all experimental sample measurements.

### HSV-2–neutralizing antibody assay.

The HSV-2–neutralizing assay was adapted from Baccari et al. (38) Briefly, HaCaT keratinocytes (Addex Bio) were seeded in 96-well plates 24 hours prior to infection with HSV-2/Gal, which expresses β-gal in an amount that is proportional to the infection. The next day, HSV-2/Gal at a MOI of 0.5 was mixed with plasma or IgG samples serially diluted in growth media with 2% FBS, and the mixture was incubated for 1 hour at 37°C. HaCaT growth medium was replaced with virus-plasma or virus-IgG mixtures afterwards and incubated at 37°C for 20 hours. Cells were subsequently lysed and incubated with a solution containing 0.5 mM chlorophenol red-β-d-galactopyranoside (MilliporeSigma), 70 mM Na_2_HPO_4_, 31.6 mM NaH_2_PO_4_, 10 mM KCl, 2 mM MgSO_4_ and 20 mM 2-mercaptoethanol for 60 minutes at 37°C. A colorimetric readout of β-gal activity was then measured on a SpectraMax M2 microplate reader (Molecular Devices). The 50% neutralizing titer (NT) against HSV-2 was defined as the reciprocal of the dilution for which the virus infectivity was reduced by 50% relative to control infection without plasma or antibodies and was calculated using the formula: [(average OD of “cells+virus” control – average OD of “cells-only” 342 control)/2 + (average OD of “cells only” control)]. The 50% NT for each test sample was interpolated by calculating the slope and intercept using the last dilution, with an OD below the 50% neutralization point and the first dilution with an OD above the 50% neutralization point. The following calculation was then used to determine the 50% NT: 50% NT = (50% neutralization point – intercept)/slope.

### HSV-2 ADCP assay.

The ADCP assay was performed as previously described (39). Briefly, gD2 protein was biotinylated using a sulfo-NHS-LC biotin (Thermo Fisher Scientific) according to the manufacturer’s instructions. Unreacted biotin was removed by buffer exchange using Amicon centrifugal concentration devices (MilliporeSigma), and biotinylated antigen (2.5 μg) was incubated with 9.6 × 10^7^ fluorescent neutravidin beads (Invitrogen, Thermo Fisher Scientific) overnight at 4°C. Beads were washed and resuspended in PBS with 1% BSA (MilliporeSigma; PBS-BSA). Serially diluted plasma and control samples were prepared in a round-bottomed, 96-well plate, 1 × 10^6^ beads were added to each well, and the plate was incubated for 1 hour at 37°C. Following equilibration, 2 × 10^4^ THP-1 cells were added to each well, and the plate was incubated overnight under standard culture conditions. The next day, the plate was centrifuged, and 100 μL supernatant was replaced with 100 μL 4% paraformaldehyde. Cells were washed and analyzed on a BD FACSCanto II. A phagocytic score was determined by gating the samples on events representing cells and calculated as follows: phagocytic score = (MFI of bead-positive cells) × (frequency of bead-positive cells, %). The corrected phagocytic score was calculated on the basis of the difference between the results of the phagocytic score for each sample and the phagocytic score for the control sample containing cells treated with neutravidin beads not conjugated to an antigen.

### HSV-2 UL25 tetramer generation.

HLA class I loci were genotyped using a sequence-based typing method. The Immune Epitope Database (IEDB) was queried for known HSV-2 epitopes as well as conserved HSV-1/-2 epitopes with confirmed binding to the determined HLA class I alleles including HLA-B*14:02, which exhibits peptide binding characteristics comparable to the patient’s HLA-B*14:01. Twelve peptides were identified in the IEDB, synthesized (GenScript) with greater than 90% purity, and resuspended in DMSO. To identify recognized CD8^+^ T cell epitopes, IFN-γ ELISPOT assays using PBMCs were performed as described previously (40). Of the peptides tested, one (DRLDNRLQL) elicited a positive IFN-γ response and was used for monomer generation. HLA-B*14:01/UL25_405–413_ (DRLDNRLQL) monomers were obtained from ImmunAware and tetramerized in-house according to the manufacturer’s instructions.

### Cytokine measurement.

Plasma cytokine levels were measured using a custom electrochemiluminescence multiarray assay according to the manufacturer’s protocol (Meso Scale Diagnostics).

### Statistics.

Statistical analysis was conducted using GraphPad Prism, version 9 (GraphPad Software). The frequency and percentage or the mean and SD are reported on basis of the data. A *P* value of 0.05 or less was considered statistically significant.

### Study approval.

The patient provided written informed consent prior to enrollment in this study, which received Emory University IRB approval (IRB00057236).

## Author contributions

AC conceptualized the study, designed and performed the experiments, performed the clinical data abstraction, provided patient samples, analyzed the results, generated the figures, and wrote the manuscript. AMS designed and performed experiments, analyzed the results, generated the figures, and wrote the manuscript. AW, DLJ, MC, MLH, and HX performed the experiments. KRJ, PR, and ALG performed experiments and analyzed the results. JLK, and JBC provided patient samples and clinical support, analyzed the data, and contributed to writing of the manuscript. DMK, LC, CRF, and RA conceptualized the study, analyzed the data, provided funding, and wrote the manuscript.

## Supplementary Material

Supplemental data

ICMJE disclosure forms

## Version 1 07/21/2022

In-Press Preview

## Version 2 09/15/2022

Electronic publication
