# Supplementary material for: Herpes simplex virus lymphadenitis is associated with tumor reduction in a patient with chronic lymphocytic leukemia
Source: J Clin Invest. 2022 Sep 15;132(18):e161109. doi: 10.1172/JCI161109 (PMC9479599; doi:10.1172/JCI161109)
Supplement: Supplemental data [file jci-132-161109-s020.pdf]

Supplementary Appendix

Table S1. Antibodies used in this study.

Antibody	Clone	Company
BCL-2	100	Biolegend
CCR7	150503	BD
CD127	A019D5	Biolegend
CD19	HIB19	Biolegend
CD19	SJ25C1	BD
CD20	2H7	Biolegend
CD28	CD28.2	Biolegend
CD3	UCHT1	Biolegend
CD3	HIT3a	Biolegend
CD38	HIT2	Biolegend
CD4	RPA-T4	Biolegend
CD45RA	HI100	Biolegend
CD5	UCHT2	Biolegend
CD71	CY1G4	Biolegend
CD8	RPA-T8	Biolegend
CTLA-4	BNI3	Biolegend
Gzmb	GB11	BD
HLA-DR	L243	Biolegend
IgD	IA6-2	Biolegend
Ki-67	B56	BD
PD-1	EH12-2H7	Biolegend
Perforin	dG9	Biolegend

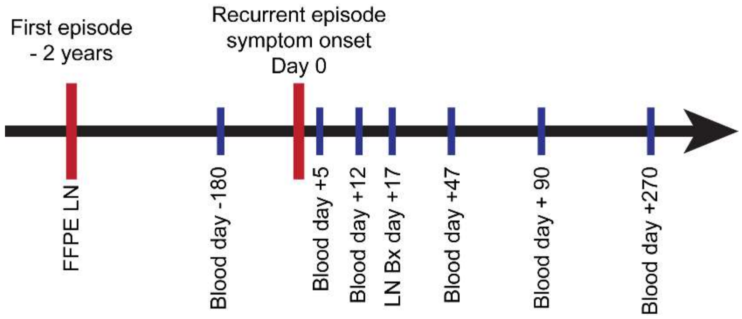

Figure S1. Timeline of samples collected and analyzed in this study.

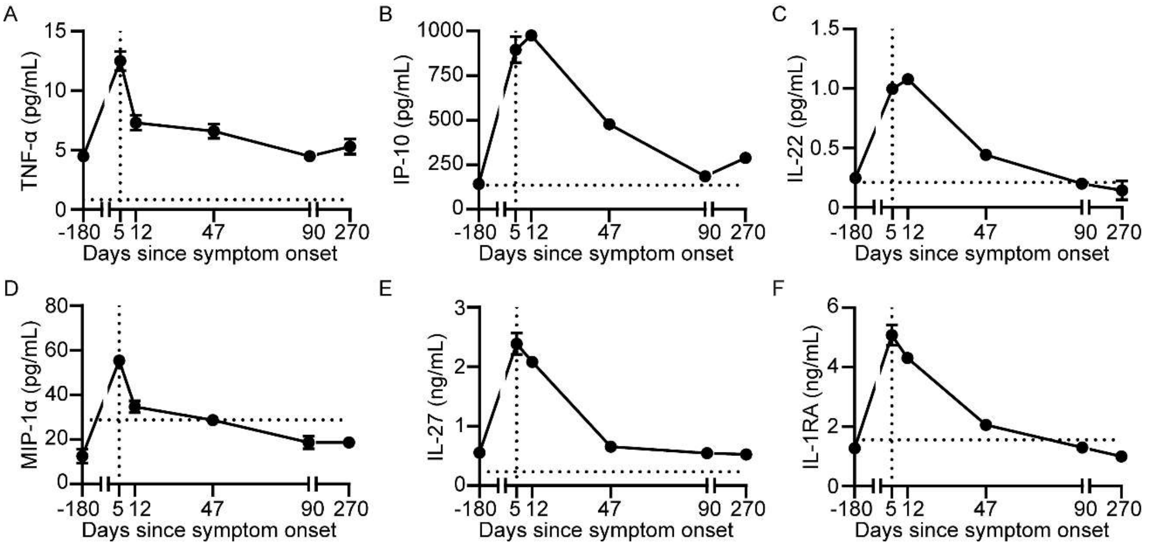

Figure S2. Mean plasma levels of additional cytokines of interest over time. Levels of TNF-α (**A**), IP-10 (**B**), IL-22 (**C**), MIP-1α (**D**), IL-27 (**E**), and IL-1RA (**F**) over time are depicted. Horizontal dotted line = levels in pooled plasma from aged-matched healthy individuals. Vertical dotted line = day of presentation. All measurements performed in quadruplicate. Error bars = standard deviation.

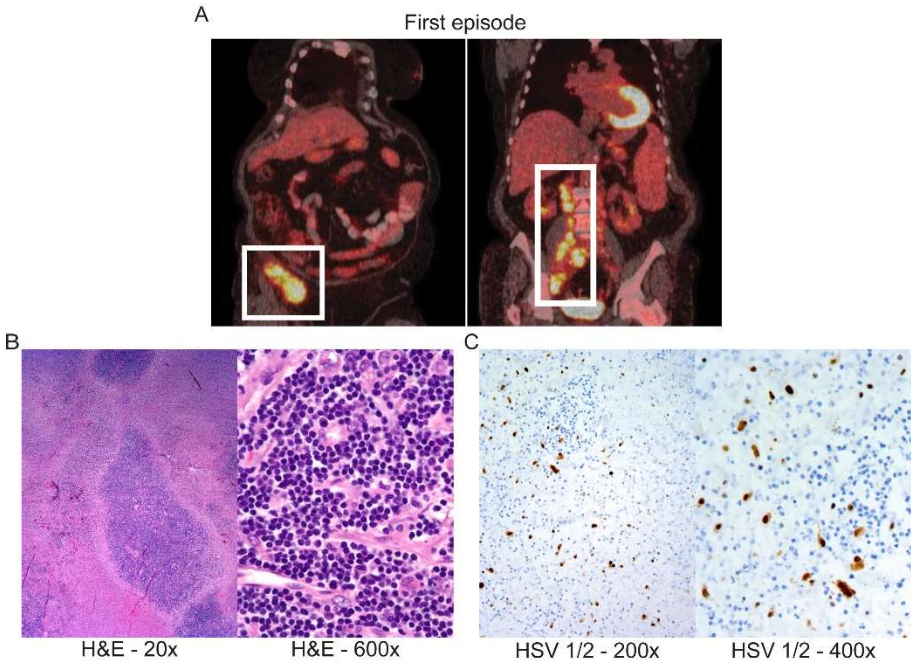

Figure S3. Clinical diagnosis of initial episode of HSVL. (**A**) PET/CT scan obtained on first episode with right-sided FDG-avid adenopathy. Micrographs of H&E (**B**) and HSV 1/2 (**C**) stains of excisional biopsy of the FDG-avid right obturator node on first presentation.
